# Ninja Warrior Athletes Have Diverse Athletic Backgrounds, High Fitness Levels, and Are Prone to Upper Extremity and Ankle Injuries

**DOI:** 10.1002/ars2.70151

**Published:** 2026-11-15

**Authors:** Carson Huntington Gardner, James Guido DiStefano

**Affiliations:** ^1^ San Francisco Orthopaedic Residency Program UCSF Health—Stanyan Hospital San Francisco California U.S.A.; ^2^ Department of Orthopaedic Surgery, San Francisco Orthopaedic Residency Program Alameda Health System Oakland California U.S.A.

## Abstract

**Purpose:**

To characterize Ninja Warrior athletes’ level and frequency of participation, exercise habits and capabilities, prior sports participation, injuries frequently sustained, and management of those injuries.

**Methods:**

A survey was available to Ninja athletes at 5 gyms and competitions between July 2023 and February 2026. Athletes were included if they were over 18 years old and competed or trained on Ninja obstacles. The survey evaluated athletic background, injury data, and injury management data. Statistical significance was determined between men and women, and a regression analysis was performed.

**Results:**

A total of 101 surveys were completed (64 men, 37 women). The mean age of respondents was 33.8 years (range 18‐57 years). Men were more likely to sustain injuries than women (incidence rate ratio 2.5). Athletes had an average body mass index of 23.2 and averaged 20.9 pull‐ups, a 195.5‐s dead hang, and an 11.0‐ft lache. The most common traumatic injury sites were hand (skin tears, 67.3%), ankle (48.5%), and shoulder (40.6%). The shoulder and elbow were the most common sites of overuse injuries. The rate of return to full preinjury level of participation was 70.7% in this study.

**Conclusions:**

Ninja Warrior athletes have broad athletic backgrounds and display high reported levels of physical fitness. Unique traumatic injury mechanisms include static and dynamic stress on the fingertips as well as eccentric loading of joints. Men, on average, sustained more injuries than women. Overuse injuries most commonly occur in the shoulder and elbow regions.

**Clinical Relevance:**

This study provides useful data about the common phenotype of Ninja Warrior athletes and the injuries they sustain, which helps to guide diagnosis and treatment of these athletes.

## Graphical abstract

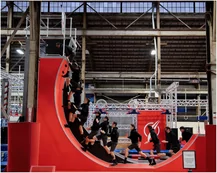

Ninja Warrior is an athletic competition consisting of a series of obstacles that must be completed sequentially. Athletes compete based on time and/or the number of obstacles completed in the course. The sport originated in Japan as a reality television competition called *Sasuke* in 1997, and a spinoff show, *American Ninja Warrior*, premiered in the United States in 2009.^1^ It has since grown in popularity with numerous Ninja‐specific gyms, leagues, and competitions throughout the world, and it is even being implemented in physical education classrooms.^1^, ^2^ Popular leagues include the World Ninja League, Ultimate Ninja Athlete Association, Ultimate Ninja Enterprises, Ninja Sports Championship, and Ultimate Backyard Warrior. In addition, the Union Internationale De Pentathlon Moderne voted to replace equestrian with a Ninja Warrior‐inspired obstacle race as the modern pentathlon's fifth discipline, which will be seen for the first time in the 2028 Olympics in Los Angeles.^3^

Ninja obstacles test a combination of upper‐body and lower‐body coordination and strength. The design for each obstacle includes elements from a variety of sports, such as gymnastics, trampoline, trapeze, climbing, and free running. Each section of the course includes a starting platform, obstacle section, and ending platform. Iconic obstacles include the salmon ladder, flying bar, cliff hanger, warped wall (Figure 1), and jumping spider as well as balance obstacles such as block run and running across spinning logs. Given the diverse nature of the sport, it is hypothesized that the injuries sustained by Ninja athletes may reflect a combination of those commonly sustained in the constituent athletic motions as well as others that are unique to the sport. Previous literature reporting injuries on obstacle courses has focused on military‐type courses or those seen in Spartan Races or Tough Mudder competitions, which feature less gymnastic and acrobatic obstacles.^4^, ^5^, ^6^ Ninja obstacles present unique and complex challenges, and there is a paucity of literature reported on the injuries sustained by these athletes. The purpose of this study was to characterize Ninja Warrior athletes’ level and frequency of participation, exercise habits and capabilities, prior sports participation, injuries frequently sustained, and management of those injuries. We hypothesized that injuries in these athletes would include those associated with falls, such as ankle sprains, as well as those caused by repetitive gripping and eccentric loading of upper extremity muscles, such as finger flexor injuries and biceps tendon ruptures.

**FIGURE 1:**
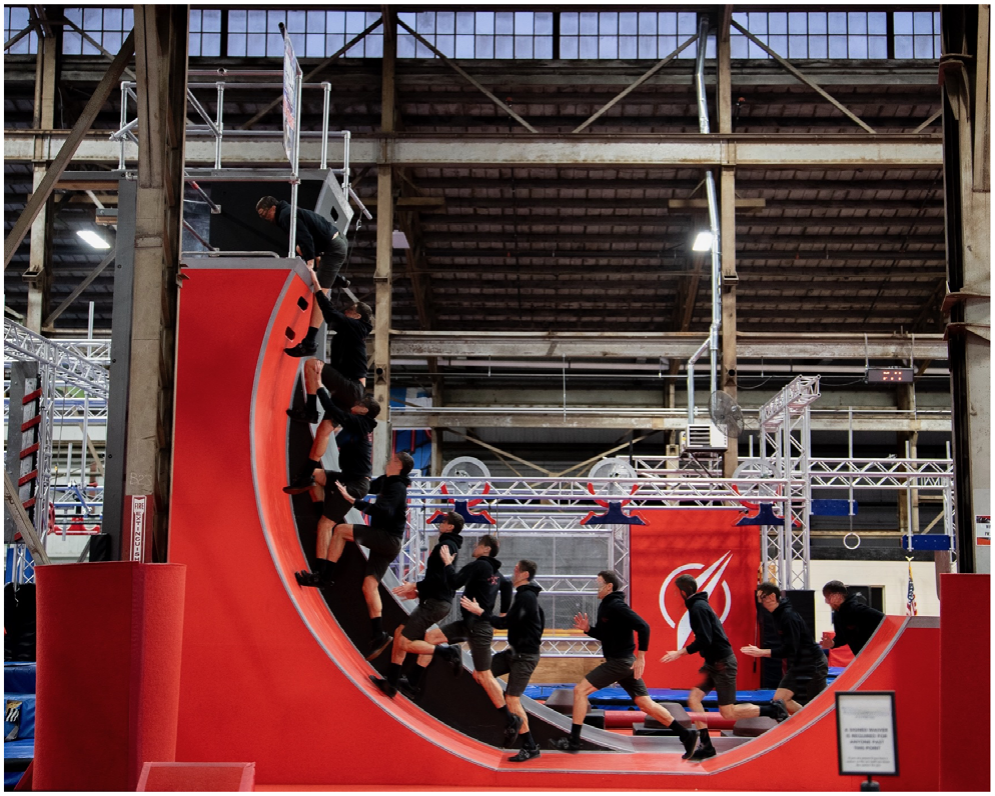
Time lapse example of an athlete completing the iconic “warped wall” obstacle. The objective is to run up the wall and then pull oneself to the top of the platform.

## METHODS

After Institutional Review Board approval (IRB18‐11211A: “Injuries Sustained by the Ninja Warrior Athlete”), a survey link was posted at 5 Ninja Warrior gyms and was distributed at Ninja league competitions between July 2023 and February 2026. Athletes were included in the study if they were over the age of 18 years, competed or trained on Ninja Warrior obstacles, and completed the survey. Athletes were excluded if they were under age 18 or did not complete the survey. The survey was created using SurveyMonkey (San Mateo, CA), and information was collected on respondent demographics, physical attributes (age, height, and weight), and secondary athletic participation, as well as the duration, frequency, and level of competition of Ninja Warrior activities. Data regarding the athletic capacity of each athlete was also obtained including the number of consecutive pull‐ups, static bar dead hang time, and maximum distance that the athlete can lache (swinging and releasing from 1 bar and catching another bar some distance away with the hands only) (Figure 2). Respondents were asked to list which areas of the body had sustained injuries and were also provided a free‐text area to describe their specific injuries. Separate sections were provided for traumatic and overuse injuries, and each was broken down into the different anatomic regions of the body (i.e., shoulder, upper arm, elbow, forearm, and wrist). Lastly, the athletes were asked if they sought medical evaluation, were formally diagnosed, and what treatments they received.

**FIGURE 2:**
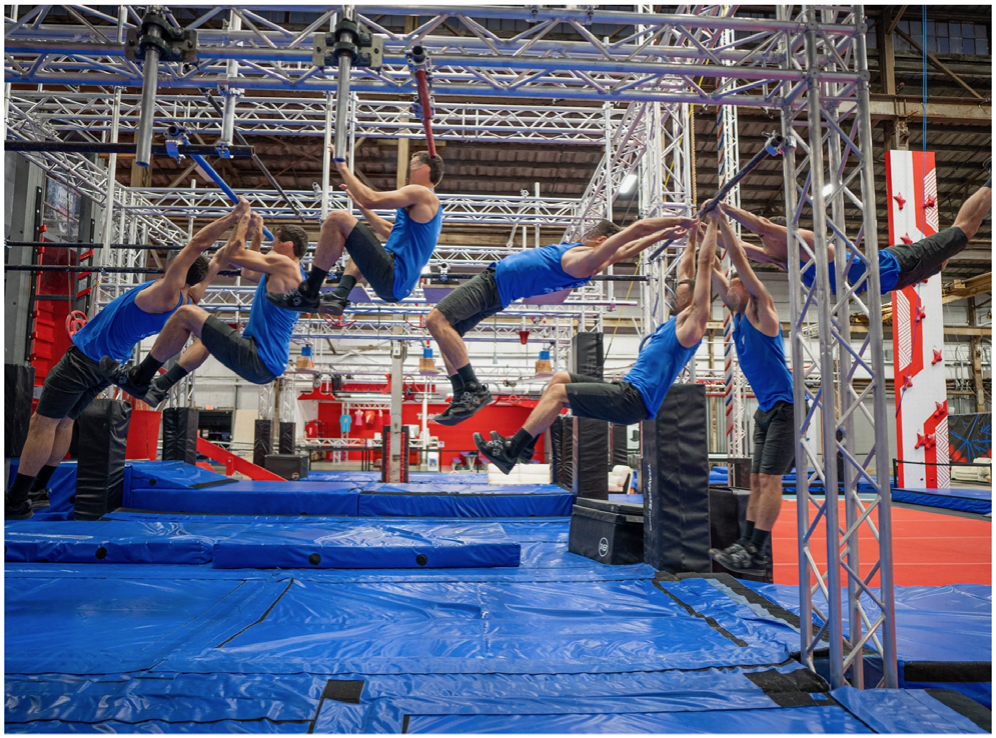
Time lapse example of an athlete performing a standard lache between 2 static bars. The lache is a fundamental skill for Ninja Warrior athletes, which can be applied when taking off or landing across a variety of static and dynamic holds.

### Statistical Analysis

Statistical significance for mean values between men and women was calculated using Welch's *t* test. Significance between men and women for the distribution of categorical variables was calculated using a combination of Pearson *χ* ^2^ test with Cramer's V and Fisher's exact test. A Poisson regression was used to determine which independent variables correlated with the number of injuries sustained. The significance was set at *P* < .05.

## RESULTS

A total of 101 Ninja athletes completed the surveys (Figure 3). There were 64 men and 37 women. The mean age of respondents was 33.8 years (range 18‐57 years) (Table 1). Of the athletes, 15.8% had been participating for less than 1 year, 7.9% had been competing for 1 to 3 years, 19.8% had been competing for 3 to 5 years, and 49.5% had been competing for more than 5 years. In all, 83 (82.2%) respondents reported having participated in an obstacle‐type competition, with the most represented being the World Ninja League (69.3%) and the TV show American Ninja Warrior (54.5%). The most common activity that athletes had participated in prior to Ninja Warrior was rock climbing (75.8%), followed by weightlifting (73.3%) and running (71.7%) (Figure 4).

**FIGURE 3:**
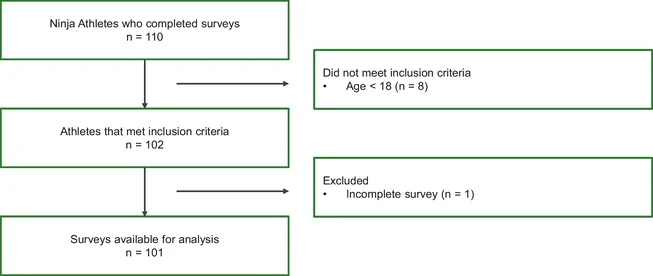
Inclusion flowsheet of 110 surveys completed, excluded (9), and ultimately included for analysis (101).

**TABLE 1:** Demographic Characteristics and Fitness Performance of Survey Respondents

	**Total**	**Men**	**Women**	***P*** **Value**
Total number of respondents, %	101	64 (63.4)	37 (36.6)	
Age, yr	33.8	35.3	31.8	.110
Height, inches	68.2	69.9	65.2	<.001
Weight, lbs	154.0	166.3	132.7	<.001
BMI	23.2	21.9	23.9	.001
Pull‐ups	20.9	25.2	13.7	<.001
Dead hang time, s	195.5	222.2	153.5	.04
Lache distance, ft	11.0	11.8	9.4	<.001
Number of major injuries	3.8	4.92	1.94	.006

BMI, body mass index; s, second; yr, years.

**FIGURE 4:**
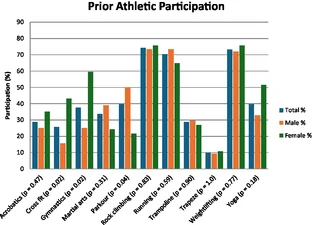
Common sports previously practiced by Ninja athletes and the percentage of respondents that reported participation. Statistical significance of the difference between men and women is denoted by the *P* Value listed, with *P* < .05 indicating significance.

Among study respondents, the average number of workouts per week was 4.8%, and 75.2% either usually or always did some type of warm‐up to begin their workout. On average, these athletes could perform 20.9 pull‐ups, a 195.5‐s dead hang, and an 11.0‐ft lache (Table 1).

The mean number of traumatic injuries leading to lost training time sustained by each athlete was 3.8 (range 0‐25). Men had a significantly higher average number of injuries sustained (4.92 vs 1.94, *P* = .006). Regression analysis accounting for years of experience and training frequency showed a 2.5 incidence rate ratio for injury for male sex (Table 2). The most common injury reported leading to lost training time was skin tears of the hand (67.3% of respondents), which occurred so frequently that many of the competitive Ninja athletes had sustained more than they could count. The percentage of athletes reporting injuries by body part, as well as the difference between men and women, is displayed in Figure 5. The second most injured body part was the ankle (48.5%), followed by the shoulder (40.6%) and fingers (34.7%). Men were more likely to sustain injuries at the ankle, finger, low back, and head/scalp (Figure 4). A list of specific injuries reported by participants is shown in Table 3. The most common site of overuse injury was the shoulder, present in 65.3% of those reporting overuse injuries (Table 4). The next most common site of overuse injury was the elbow/forearm (54.7%).

**TABLE 2:** IRR of Injury Based on Various Athlete Traits, Demographics, or Training Patterns

**Predictor**	**IRR (95% CI)**	***P*** **Value**
Age	1.02 (0.98‐1.05)	.328
BMI	0.95 (0.85‐1.06)	.342
Pull‐ups	0.99 (0.95‐1.03)	.56
Sex (male)	2.50 (1.23‐5.07)	.**011**
Years of experience	1.05 (0.81‐1.37)	.721
Warm‐up frequency	0.95 (0.66‐1.36)	.776
Training sessions, wk	1.37 (0.99‐1.90)	.060
Workouts, wk	1.04 (0.87‐1.23)	.693
Number of athletic backgrounds	0.94 (0.79‐1.11)	.473
Has competed	1.25 (0.59‐2.67)	.556

*Note*: Bold value indicates statistical significance (*P* = .011).

BMI, body mass index; CI, confidence interval; IRR, incidence rate ratio; wk, weeks.

**FIGURE 5:**
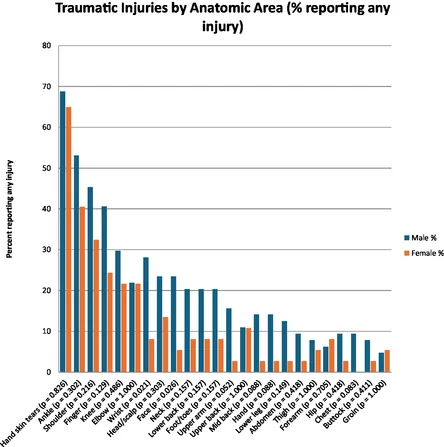
Most common body parts injured and the percentage of athletes reporting a history of at least 1 injury in the given body part. Statistical significance on the difference between men and women is denoted by *P* Value, with *P* < .05 denoting statistical significance.

**TABLE 3:** List of Most Common Injuries Reported by Survey Respondents

**Injury Sustained**	**Number of Times**
Skin tear (hand)	>100*
Ankle sprain	30
Finger/hand fracture	11
Rotator cuff tear	9
Concussion	9
Finger sprain	8
Labrum tear (shoulder)	7
ACL tear	6
Flexor pulley injury	6

*Note*: The following injuries were reported fewer than 6 times: meniscus tear (5), wrist sprain (5), ankle fracture (4), broken nose (4), facial laceration (4), biceps tear (3), latissimus strain (3), shoulder dislocation (4), wrist fracture (4), Achilles rupture (3), calf strain (2), coccyx fracture (2), elbow fracture (2), tibia/fibula fracture (2), patella dislocation (2), patella fracture (2), cervical disc herniation (1), elbow dislocation (1), forearm fracture (1), femur fracture (1), labrum tear (hip) (1), pectoralis strain (1), plantaris tendon rupture (1), proximal hamstring strain (1), sternoclavicular joint sprain (1), and foot/toe fracture (2).

ACL, anterior cruciate ligament.

*Skin tears of the hand were reported as being so frequent that most athletes are unaware of how many they have had. They were the most frequently reported injury.

**TABLE 4:** Most Common Overuse Injuries Reported by Athletes

**Body Part**	**Number Reporting Overuse Injury (%)**	**Male, %**	**Female, %**	***P*** **Value**
Shoulder/arm	49 (65.3)	54%	42%	.47
Elbow/forearm	41 (54.7)	49%	28%	.13
Wrist/hand	23 (30.7)	30%	11%	.12
Hip	2 (2.7)	2%	3%	1.0
Thigh	5 (6.7)	6%	3%	.77
Knee	16 (21.3)	19%	11%	.51
Shin	8 (10.7)	11%	3%	.47
Foot/ankle	19 (25.3)	22%	14%	.51

Injured athletes were most likely to use rest (94.1%) and ice (95.3%) to help treat their injuries, and 79.1% also used non‐steroidal anti‐inflammatory drugs (Table 5). The most frequently visited health care provider was a physical therapist (54.7%), followed by orthopaedic surgeon (36.0%), urgent care (32.6%), and primary care (22.1%). Of the athletes, 70.7% reported full return to preinjury status, whereas 26.8% returned to a modified or lower‐level status and 2.4% were unable to return to sport.

**TABLE 5:** Most Common Self‐Treatment Employed by Injured Athletes

**Home Treatments Used**	**Number of Respondents (%)**	**Male, %**	**Female, %**	***P*** **Value**
Rest	81 (94.1)	87%	72%	.18
Ice application	82 (95.3)	84%	81%	.78
Heat application	50 (58.1)	52%	47%	.77
Compression	56 (65.1)	57%	56%	.77
Elevation	61 (70.9)	62%	61%	.97
Over‐the‐counter anti‐inflammatory medication (Ibuprofen, Naproxen, etc.)	68 (79.1)	68%	69%	.96
Over‐the‐counter pain medication (Tylenol)	38 (44.1)	38%	39%	.97

## DISCUSSION

The most important finding of this study was that Ninja Warrior athletes sustain a variety of traumatic and overuse injuries despite a high level of physical fitness and regular participation in the sport. The most common athletic endeavor prior to Ninja Warrior was rock climbing, which encompasses several similar skill sets to those required to be a competitive obstacle athlete. The next 2 most common athletic endeavors prior to Ninja Warrior were running and weightlifting, which is not surprising given their fundamental role in sports and the athletic level of most regular participants. The respondents also reported high rates of participation in other niche sports that translate closely to Ninja Warrior skills, such as parkour (40.8%), gymnastics (38.8%), and acrobatics (30.2%).

The most common injury locations reported leading to lost training time in our study were skin tears of the hand (67.3%), ankle (48.5%), shoulder (45.3%), and fingers (40.3%). The most common site of overuse injury was the shoulder (65.3%), followed by the elbow/forearm (54.7%). A similar study was published by Lindner et al. in March 2026 looking at the epidemiology of injuries in 103 Ninja Warrior athletes.^7^ They did not differentiate traumatic and overuse injuries but found that the most impacted joints were the shoulder (19.1%), followed by the ankle (12.6%) and then wrist (11.5%). They also found an association between body mass index, visual analog scale score, and 5 or more training sessions per week and athlete return‐to‐sport levels.

In the present study, the only statistically significant predictor of injury risk was male sex (*P* = .006). This finding persisted in the Poisson regression analysis while accounting for the frequency of training, previous years of training, and a history of competing. Men had significantly longer average lache distance (11.8 vs 9.4 ft) and were also heavier on average (166.3 vs 132.7 lbs). A greater force must be generated to propel a larger mass over a longer distance, and this would correspond to more stress on joints because they are hanging from, grasping, and transferring between obstacles.

We noted that athletes frequently reported a variety of hand/finger injuries such as skin tears, flexor pulley injuries, and hand/finger fractures. Overall, the most frequent injury reported was lacerations/abrasions of the skin of the hand. This has been reported in other sports that require repetitive gripping with the hands such as rock climbing,^8^, ^9^ gymnastics,^10^, ^11^ and weightlifting.^12^, ^13^ Ninja participation includes swinging and releasing from a variety of bars, small ledges, and other hand holds of various sizes and contours that place significant force on the hands. The obstacle designs where athletes must carry the full weight of the body through the grip or fingertips put them at risk of a variety of hand injuries. A study by Logan et al. found that flexor tendon injuries were the most common injury in climbers, followed by hand abrasions/lacerations.^9^ Combining flexor tendon and flexor pulley injuries into a single category would make them the third most frequent injury in this cohort, behind hand skin injuries and ankle sprains. The varied providers who care for these athletes should be aware that flexor tendon and flexor pulley injuries are complex problems that warrant referral to a hand surgeon for evaluation and management.^14^, ^15^

The second most common area injured in the population of our study was the ankle. Ankle injuries are frequently cited as one of the most common injuries in gymnastics as well as traditional military style obstacle courses.^5^, ^10^, ^11^, ^16^, ^17^ In the sport of Ninja, athletes are at risk of sustaining rotational ankle injuries when running across balance obstacles or when dismounting from upper‐body obstacles during the landing. There is also a particular vulnerability for Achilles tendon ruptures when running up the sloped warped wall (Figure 3), and we identified 3 athletes that sustained Achilles ruptures in this manner. The warped wall requires forceful plantarflexion out of a highly dorsiflexed position, which puts significant strain across the tendon. An additional opportunity for injury occurs if the athlete has failed the attempt and catches the toe of their shoe when dropping or sliding down the wall.

There was a high level of competitive athletes in this cohort, with 60.3% competing in the World Ninja League and 54.5% having competed on the TV show American Ninja Warrior. A subgroup of about 25% of the study population only warmed up sometimes, rarely, or never, and this was associated with approximately 18% of the respondents who had not participated in any formal obstacle competition. A wide range of prior literature has shown that athletes with lower levels of training and conditioning, or with less thorough warm‐up routines, tend to have a higher propensity for injury.^18^, ^19^, ^20^, ^21^, ^22^ Medical providers as well as Ninja gyms should be cognizant of the different levels of participants in Ninja activities and the variable injury risk they may incur. Gyms may help to protect novice participants from injury through instructive warm‐up routines, training, and conditioning programs.

Overuse injuries were most reported at the shoulder and elbow, likely reflecting the frequency with which participants hang and swing from obstacles. Medial epicondylitis is a frequent overuse injury seen in climbing, which is also common in Ninja athletes.^23^ Repetitive loading, particularly eccentric loading of the forearm flexors through forearm pronation, wrist flexion, and finger flexion, leads to pain at the flexor origin at the medial epicondyle.^23^ Athletes frequently reported shoulder pathologies including rotator cuff tendonitis, subacromial impingement, and shoulder instability. Multidirectional instability is one of the most frequently cited chronic shoulder pathologies in female gymnasts and should be considered for Ninja athletes with recurrent shoulder pain, instability, or a history of ligamentous laxity.^23^, ^24^

The cohort in this study reported 6 concussions and a single cervical spine injury, both of which have high potential morbidity. Previous literature focused on trampolines has reported high rates of head and neck injury.^25^ Concussions in gymnastics are managed stringently given the high vestibular demands of the sport, and watchful concussion protocols are also recommended for Ninja athletes.^17^, ^26^

The return‐to‐play rate in the study cohort was relatively low, with only 70.7% reporting a full return to preinjury level after their cumulative injuries. The low return to play may reflect a high degree of injury, intense demands of the sport, and hesitancy to seek formal medical care. Nearly all athletes reported appropriate conservative management of injuries including rest, ice, NSAID use, and compression. However, only about half of respondents were evaluated by a physical therapist (54.7%), and 36.0% were seen by an orthopaedic surgeon. A potential explanation that athletes seldom seek evaluation from orthopaedic surgeons is the lack of side‐line coverage or because access to primary care doctors or therapists is more available. Given that 29.3% of the athletes in this study had not achieved a full return to preinjury activity level, more focus should be placed on encouraging athletes to seek medical attention to properly evaluate injuries and coordinate treatment plans. Improving physician or trainer coverage of competitions could also lead to earlier diagnosis and may aid in increasing the percentage of athletes that return to their prior level of play after injury.

The sport of Ninja Warrior is growing rapidly both in the United States and internationally.^1^, ^2^ With a Ninja‐inspired obstacle course being incorporated in the 2028 Olympics, it is important for health professionals to understand the biomechanical stress that the obstacles commonly exert on competitors.^3^ This will allow physicians, trainers, and other medical staff involved in caring for obstacle athletes to better prepare for and treat the spectrum of commonly occurring injuries and help develop injury prevention programs.

### Limitations

There are multiple limitations to the study. The data was collected retrospectively and was self‐reported by survey participants, potentially leading to inaccurate diagnosis reporting as well as over‐ or under‐reporting of injuries (recall bias). Although there are certain common features to Ninja obstacles across gyms, there are many unique variations and designs to the obstacles present at the individual gyms that are not fully encompassed with the study. The survey was distributed by being posted in multiple gyms with a QR code, limiting the ability to calculate the survey response rate. Our results are subject to volunteer bias. The survey was also filled out only by injured athletes, and injury reporting was not confined to a certain timeframe, thus limiting any conclusions which can be drawn about the incidence of specific injuries.

## CONCLUSIONS

Ninja Warrior athletes have broad athletic backgrounds and display high reported levels of physical fitness. Unique traumatic injury mechanisms include static and dynamic stress on the fingertips as well as eccentric loading of joints. Men, on average, sustained more injuries than women. Overuse injuries most commonly occur in the shoulder and elbow regions.

## DISCLOSURES

The authors (C.H.G., J.G.D.) declare that they have no known competing financial interests or personal relationships that could have appeared to influence the work reported in this article.
